# Rodent models for psychiatric disorders: problems and promises

**DOI:** 10.1186/s42826-020-00039-z

**Published:** 2020-04-15

**Authors:** Matthew Baker, Sa-Ik Hong, Seungwoo Kang, Doo-Sup Choi

**Affiliations:** 1 Department of Molecular Pharmacology and Experimental Therapeutics, Mayo Clinic College of Medicine, 200 First Street SW, Rochester, MN 55905 USA; 2 Neuroscience Program, Rochester, MN USA; 3 Department of Psychiatry and Psychology, Mayo Clinic College of Medicine, Rochester, MN USA

**Keywords:** Rodent models, Psychiatric disorders, Circuits, Genetics, Behaviors

## Abstract

Psychiatric disorders are a prevalent global health problem, over 900 million individuals affected by a continuum of mental and substance use disorders. Due to this high prevalence, and the substantial direct and indirect societal costs, it is essential to understand the underlying mechanisms of these disorders to facilitate development of new and more effective treatments. Since the advent of recombinant DNA technologies in the early 1980s, genetically modified rodent models have significantly contributed to the genetic and molecular basis of psychiatric disorders. Despite significant advancements, many challenges remain after unsuccessful drug development based on rodent models. Recent human genetics show the polygenetic nature of mental disorders, identifying hundreds of allelic variants that confer increased risk. However, given the complexity of the brain, with many unique cell types, gene expression profiles, and developmental trajectories, proper animal models are needed more than ever to dissect genes and circuits in a cell type-specific manner to advance our understanding and treatment of psychiatric disorders. In this mini-review, we highlight current challenges and promises of using rodent models in advancing science and drug development, focusing on advanced techniques, and their applications to rodent models of psychiatric disorders.

## Introduction

An estimated 970 million people worldwide are affected by substance use or mental disorders. At the individual level, these psychiatric disorders were the leading cause of years lived with disability of any disease group, and were comparable to cardiovascular and circulatory diseases for disability-adjusted life years [1]. In 2010, the estimated global burden of psychiatric illness was an estimated $8.5 trillion [2]. Despite these profound individual and societal costs, substance use and mental disorders still represent a large unmet need in society. Although traditional antipsychotics and antidepressants have improved the lives of many patients, many individuals are resistant or relapse following typical treatments, with new drug development facing a multitude of challenges. Alcohol use disorder, for example, has had the only 3 FDA approved medications available for decades, with mixed effectiveness in promoting cessation of alcohol use.

These heterogeneous responses to typical treatments are only further hindered by diagnostic criteria based on symptomology, with reliable biomarkers for disease diagnosis and monitoring yet to be established. This emphasizes the importance of preclinical research and animal models of psychiatric disorders to fully characterize their underlying genetic and neural mechanisms, and facilitate the development of new treatments. In particular, rodent models have been particularly useful towards this end. Regarding genetic architecture, brain structures and behavioral phenotypes, rodent models are more similar to humans than other non-mammalian models such as *C. elegans*, *Drosophila* and zebrafish. Additionally, rodent models are cost- and time-effective compared to primate models for drug screening and development.

Despite some instances of drugs showing promise in rodents and failing human trials, these model systems are needed to untangle the complexity of the brain and its vast array of cell-types, each with unique gene expression profiles and interconnections in distinct neural circuits, ultimately giving rise to behavioral states. In this review, we focus on newly available forward and reverse genetics models and how these models are useful for neural imaging and modulation techniques, which will give researchers an unprecedented ability to understand the connection between genes, circuits, and behavior, and facilitate the development of new biomarkers and therapies for individuals suffering from substance use and other mental disorders.

### Cell-specific molecular analysis

**Fig. 1:**
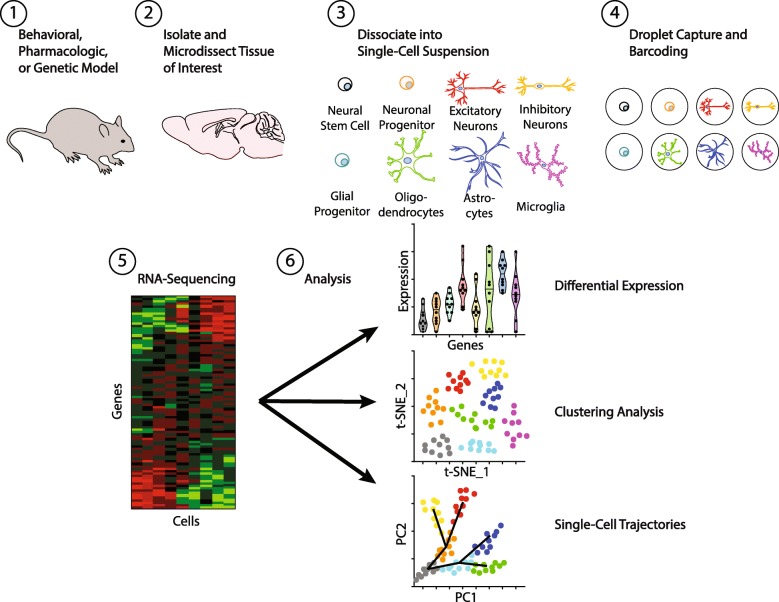
Single-Cell RNA Sequencing Workflow. Brain tissue from a behavioral, pharmacologic, or genetic mouse model can be isolated and microdissected to achieve brain-region specificity [1, 2]. Next cells can be dissociated using cell-specific markers, barcoded, and droplet captured [3, 4]. Individual cells undergo RNA-sequencing and can be analyzed using differential expression to assess genes that drive differences between cell types, treatment conditions, or cell trajectories [5, 6]. A clustering analysis can be performed to identify and group cells based on gene expression markers. Single-cell trajectories can track the genetic regulation of cell-fate decisions in pseudo-time in immature, intermediate, and mature cell-types

The genetic and molecular characterization of psychiatric disorders has drastically improved over the last decade with the development of large-scale sequencing technologies. Advances in forward genetics have allowed scientists and physicians to examine the entire genome of patients more quickly and cost-effectively than ever before. Given the profound genetic and environmental interactions in the etiology of psychiatric disorders, other next-generation sequencing technologies are also important to characterize changes in gene expression profiles associated with allelic variants or from epigenetic modifications (e.g. RNA-sequencing, epigenomics). Although blood driven DNA or RNA sequencing may provide correlational etiology, these techniques fundamentally require primary tissue from the brain, and have largely not been feasible outside of post-mortem brain samples in humans. Therefore, rodent models are particularly advantageous for measuring gene expression profiles in behavioral or genetic models of psychiatric disorders. Despite these advances, the brain is composed of thousands of different cell types with diverse gene expression profiles and developmental trajectories. Having considered these limitations, it would be particularly useful to further clarify the role of genetic risk loci for psychiatric disorders, by pinpointing unique changes in gene expression down to specific cell-types. Recently, single-cell based RNA sequencing (scRNA-seq) can more precisely address the molecular and biological basis of several psychiatric disorder phenotypes. With the recent advancement of scRNA-seq, several publically available datasets offer cell-specific profiles [3–5]. Furthermore, human [6, 7] and mouse [8] cell atlases enable investigators to validate their data and cell-types. As shown in Fig. 1, droplet capture-barcoding is a commonly used technology called “Drop-Seq” for microfluidic-based scRNA-seq [9]. Using split and pool DNA sequencing, short DNA barcode (typically 10–16 bp long) “tags” identify the origin of the cells [10] and can be analyzed for differential expression of specific genes, cell clustering, and cell trajectories, among others.

In one exemplary study, the authors investigated the neural dynamics of mouse prefrontal cortex mainly comprising of anterior cingulate, prelimbic and infralimbic areas during adolescence and in a model of addiction [11]. They sequenced approximately 30,000 cells from 12 independent biological samples, resulting in 8 major cell clusters that were detected (Similar to Fig. 1). It is also common to use cell-specific markers. The non-neuronal cells are clustered as astrocytes (Gja1+), oligodendrocyte (Aspa+), newly formed oligodendrocytes (Bmp4+), oligodendrocyte precursors (Pdgfra+), microglia (C1qa+) and endothelial cells (Flt1+). The neurons express Snap25 and can be divided into excitatory (Slc17a7+) and inhibitory (Gad2+) neurons. In this study, the authors demonstrated that the excitatory neurons form the largest (52.3%) cell class in the PFC, while the inhibitory neurons comprise a smaller portion (4.3%) of the total populations, consistent with the general excitatory/inhibitory ratio reported in most cortical areas. With these basic characteristics, they found that neuron-specific gene expression is significantly altered during adolescence (between P21 and P60) including cell type-specific regulation of genes implicated in major neuropsychiatric disorders. Also, in a chronic cocaine addiction paradigm, prolonged withdrawal was found to have a profound impact on neuron-specific gene expression.

This new technique will reveal the molecular dynamics of many psychiatric disorders. However, scRNA-seq has several notable drawbacks. First, it is still expensive to carry out large scale sequencing, especially compared to tissue-based RNA sequencing. Second, like other -omics approaches, it requires optimization of the data analysis process. Recently, a study provided a promising data analysis platform for complex traits [12]. The authors tried to integrate or align scRNA-seq data with genome-wide association studies (GWAS). Briefly, they proposed 3-step workflow to investigate associations of traits with cell types: 1) identify significantly associated cell types after correcting *P*-value across all tested cell types, 2) within the dataset, identify independent associations with step-wise conditional analysis, and 3) evaluate if the significant associations with cell-types from distinct datasets are driven by similar genetic signals. With these steps, the scRNA-seq data will be meaningfully integrated with clinical data as well.

### Precision genetic engineering approach

Many of these ‘omics’ techniques have provided large datasets, documenting hundreds of risk-conferring genes and alleles in psychiatric disorders. However, given the complex polygenetic nature of these disorders, the connection between genotype and phenotype often remains obscured. This requires further loss and gain of function studies in animal models to determine how a change in gene expression or altered gene products may contribute to the development or expression of maladaptive behavior or cognition.

Classical transgenic rodent models contribute to understanding loss of function for specific genes in psychiatric disorders using conventional KO mice. In addition, the advent of tissue-specific Cre-loxP system enables to investigate more region and temporal deletion and overexpression of gene. However, it has limitations to understand the cell-specific or circuit-specific function of certain genes. Further, the generation of lines can often be financially costly and time-consuming. Other methods such as short interfering RNA’s (siRNA’s) have also been successfully used to reduce expression of target genes, but have some limitations in its flexibility and degree of knockdown.

**Fig. 2:**
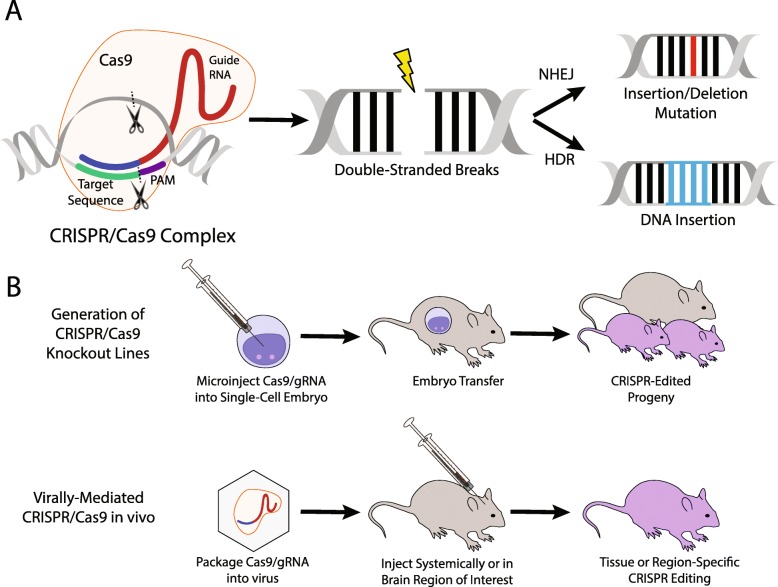
Clustered regularly interspaced palindromic repeats/CRISPR-associated endonuclease (CRISPR/Cas9) workflow. The target sequence is followed by a protospacer adjacent motif (PAM) and is targeted via designed guide-ribonucleic acid (gRNA). The Cas9 protein associates with the gRNA and creates targeted double-stranded breaks which can be repaired via the non-homologous end joining (NHEJ) or homology directed repair (HDR) pathways (A). NHEJ can result in insertion or deletion mutations, resulting in non-expressed or non-functional protein. Combined with donor deoxyribonucleic acid (DNA), HDR can reliably insert genetic material into the targeted area. CRISPR/Cas9 can be used to efficiently create mutant mouse lines by injecting gRNA/Cas9 into single-cell embryos, which are transplanted into pseudo pregnant females, resulting in mutant progeny. Alternatively, gRNA/Cas9 can be virally packaged and injected in vivo to achieve some tissue or region-specific gene editing

Recently, the clustered regularly interspaced short palindromic repeats/CRISPR-associated protein 9 (CRISPR/Cas9) system has emerged as a powerful tool, allowing researchers to edit the genome of any organism with precision [13]. Generally, the CRISPR/Cas9 functions through the induction of targeted double-stranded breaks, which are subsequently repaired through the non-homologous end joining (NHEJ) or homology-directed repair (HDR) pathways (Fig. 2A). NHEJ can result in random insertion or deletion mutation, potentially altering the reading frame or introducing early stop codons to effectively knock out a target gene. Alternatively, HDR can insert specific promoters, genes, or allelic variants using donor DNA.

While clinical trials utilizing CRISPR-based gene-editing have emerged in other fields, clinical applications for psychiatric disorders remain distant, and will require marked advancements in our understanding of how specific mutations may contribute to brain dysfunction. In rodent models, CRISPR can be used to develop knockout lines more efficiently than traditional methods by injecting single-cell embryos with Cas9 protein and guide RNA targeting the gene of interest, and re-implanting the mutant embryos back into the donor mouse. Alternatively, when packaged into a virus, CRISPR can be used to edit genomes in a region or cell type-specific fashion in vivo (Fig. 2B). One advantage of CRISPR over traditional gene knockout models is its ability to edit many genes simultaneously, which is particularly necessary considering the complex polygenetic nature of psychiatric disorders. Additionally, specific allelic variants or even human-exclusive genes identified as risk-conferring via human genetic screens can be replicated in rodent models to detect causal variants and identify underlying mechanisms of human disease.

For example, the 3q29 deletion is known to increase the risk of developing an intellectual disability, autism spectrum disorder, generalized anxiety disorder, and greater than the 40-fold increased risk for schizophrenia [14]. Utilizing a CRISPR/Cas9 system, the researchers injected the guide-RNA and Cas9 into single-cell mouse zygotes. These zygotes were implanted into pseudo-pregnant females, and the pups were screened for the deletion using PCR. These pups were then backcrossed to produce heterozygous mutants with the 3q29 deletion and assessed for a number of behavioral and developmental measures. The study found that mice harboring the 3q29 deletion displayed a number of behavioral and developmental impairments consistent with 3q29 deletion syndrome patients including social interaction, cognitive function, and reduced body weight, among others. This demonstrates the ability of the CRISPR/Cas9 system to develop rodent models based on human genetic findings and further understand the causal contributions of specific risk-conferring genetic variants.

There have been some trade-offs in editing efficiency and the degree of edit predictability between HDR and NHEJ and concerns of off-target effects and the generation of mutant proteins with unknown functions [15]. More recent studies have attempted to improve upon these genome-editing strategies using alternative repair pathways, Cas9 replacements to introduce staggered double-stranded breaks, as well as reverse-transcriptase-based systems that do not require double-stranded breaks among others [16–18]. While it is still in its infancy, it is clear that CRISPR and other gene-editing systems will play an increasingly important role in establishing causal relationships between genes and behavior and improve our understanding and treatment of psychiatric disorders.

### In vivo dynamic neural imaging technology

While uncovering the role of genetic risk factors in the development of psychiatric disorders is now more possible than ever, altered gene expression and allelic variants ultimately have their effect through directly or indirectly altering the structure or function of neurons in the brain. Further, these neurons are wired together within and/or between brain regions producing complex neural circuits that ultimately drive behavior. 

**Fig. 3:**
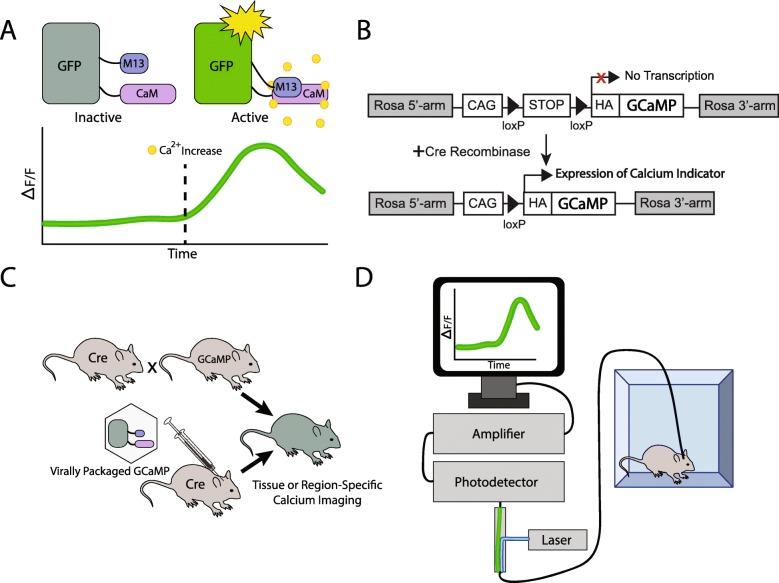
Calcium imaging workflow. GCaMP is a fusion protein composed of green fluorescent protein (GFP), calmodulin (CaM), and a peptide sequence from myosin light chain kinase (M13). In the presence of calcium, CaM undergoes a conformational change, and binds to the M13 protein, resulting in detectable fluorescence from the GFP (**A**). Cell-type specific expression of the calcium indicator, GCaMP can be achieved utilizing a cre-lox system (**B**). LoxP sites flank a stop codon, which inhibits transcription of GCaMP. In the presence of cre-recombinase, recombination removes the stop codon, permitting expression of GCaMP. This system can be achieved by crossing transgenic mice expressing cre-recombinase in a certain cell type with mice expressing cre-dependent GCaMP. Alternatively, cre-dependent GCaMP can be virally packaged and injected in vivo into mice expressing cell type-specific cre-recombinase (**C**). In fiber photometry, the fiber optic cable is utilized to deliver blue light to excite GFP, as well as detect and amplify the fluorescent signal produced in the presence of calcium (**D**). Microendoscopes (not shown) use a similar setup, but with the laser, photodetector, and amplifier mounted on top of the rodents head in addition to the imaging lenses

Traditionally, neurophysiological techniques such as electrophysiology have been used to record neural activities. Currently, many techniques enable us to assess neural activity in a real-time manner, which come with various advantages and limitations. One promising advance is calcium imaging due to its improved feasibility in imaging freely-moving animals. Calcium imaging has been a reliable and well-established tool of directly recording neural activity for decades [19–21]. Considering the fact that action potential, a general character of neurons, is evoked via the balance of rapid influx and outflux of ion, including calcium, across the cytoplasmic membrane [22, 23], intracellular changes in calcium concentration can be treated as a signal for cellular excitation and action potential formation [24]. Calcium indicators consist of circularly-permutated GFP, calcium binding proteins-calmodulin (CaM) and calcium/calmodulin-binding peptide derived from skeletal muscle myosin light chain kinase, thus allowing to measure the changes in its fluorescence intensity in response to intracellular changes (Fig. 3A) [25]. Moreover, the creation of genetically encoded calcium indicators (GECIs), combined with technical advances in viral-mediated gene transfer and transgenic animal availability, has provided fine expression of the calcium indicators in cell-type and circuit dependent manners (Fig. 3B-C) [26–28]. Among several types of GECIs, the most commonly used calcium indicators are the GCaMP family [25]. The GCaMPs are categorized by its generation (1 to 7) and characteristics such as temporal resolution, brightness, signal-to-noise ratio, and fluorescent probes [29–31]. Because of the dramatic improvement in temporal resolution and signal-to-noise ratio, the imaging technology has been adapted for use in freely-moving animals, which extend the application of in vivo calcium imaging [32].

Recently, several applicable options of in vivo calcium imaging are available, according to the primary experimental goals. Largely, optic fiber-based and probe-based observations of GECI fluorescence such as fiber photometry and micro-endoscopy are currently available approaches. The fiber photometry is used to observe regional activity by exciting GECI and measuring the changes in fluorescence utilizing chronic implantation of optic fiber, ranging from 200 to 400 μm in diameter (Fig. 3D). This assessment has a similar surgical process to optogenetics and is presumed to reflect the summed neural activity within the entire cellular population in the target brain region [33, 34]. Since the light sources and optic fibers are similar to those that are used for optogenetics and it is possible to measure the several brain regional activities with branched fibers, this method has advantages of relatively easy to perform stereotaxic surgery for implantation and data analysis [35]. However, as mentioned above, fiber photometry integrates all the observed photons and only provides bulk-light information made by the entire GECIs.

To visualize individual cellular dynamics, the advent of head-mountable micro-endoscopic techniques has been quite useful. Observation of GECIs with a single-cell resolution has required a high enough resolution, a path of fluorescence light transfer from deep brain, and use of a full-size microscope, which traditionally prevents the use of free-moving animals. In addition to dramatic improvements in GECIs, new development of miniaturized micro-endoscopes are able to be mounted on the head of rodents with light-burden of weight (approximately 2 g) and allows for single-cell resolution in behavior-synchronized observation. This miniature microscope typically has fluorescence excitation light source and sensor within the head-mounted part and read the GECIs’ value via chronically implanted GRIN lenses (250 to 1000 μm in diameter). Since this head-mount micro-endoscopic approach can detect changes in GECIs at a single-cell resolution level with spatial information, it is possible to observe whether there is a specific colony of cells that is responsive to specific behavioral patterns or emotional states. In addition, monitoring the spatial location of individual cells makes it possible to trace an identified cell across multiple recording sessions. The main limitation of this approach so far is that the use of single-photon microscopy does not provide the rejection of fluorescence reflecting the outside of the focal plane. Thus, to observe the changes in detailed compartments of cells, further computational process is essential to reduce the noise in the data. To overcome the limitation of this head-mount single-photon micro-endoscopy, along with the development of two-photon head-mountable microscopes [36] new two-photon table-top microscopic approaches have developed to measure animal behavioral navigation with 3D virtual reality environments. For example, a 3D virtual reality behavioral platform for open field can provide a scene that can be changed by the paws’ movements of awake and head-fixed rodents to two-photon table-top microscope [37, 38].

Although we focused on calcium imaging in this review to explain the approaches of measuring neuronal activity synchronized with preclinical animal behaviors, there are also several approaches to visualize spatiotemporal cellular dynamics using fluorescence indicators targeting a wide array of cellular activity such as sensing chloride ion and voltage changes [39–42].

### Cell- and circuit-specific neural manipulation

In conjunction with neural imaging, manipulation of specific neural circuits is necessary to establish a causal role of specific neural circuits resulting in behavior or symptoms related to a psychiatric disorder. Traditional methods in rodent models involved lesioning brain regions, electrical stimulation, or pharmacology. However, given the vast diversity of cell types and intricate neuronal connections within and between brain regions, these methods can often be crude, unable to pinpoint the exact neural structures involved. Recently, increasingly powerful tools such as chemogenetics and optogenetics have allowed for modulation of neural activity with high temporal and cell/circuit-specificity.

**Fig. 4:**
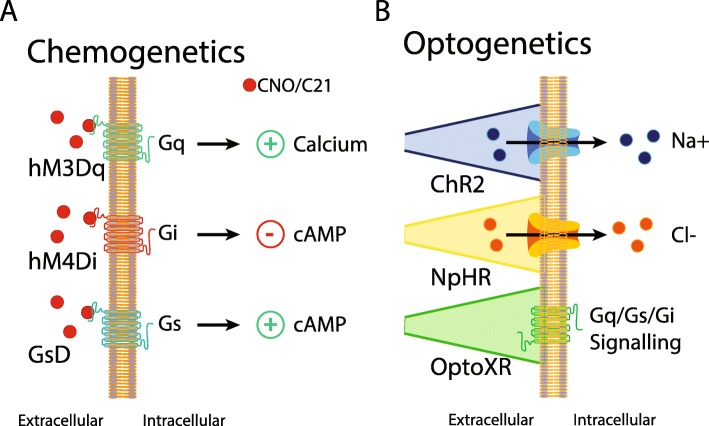
Cell-type specific neuromodulation can be achieved using specialized ligand-activated or light-activated receptors. Chemogenetics (**A**) involves using designer receptors exclusively activated by designer drugs (DREADDs) which are generally modified human muscarinic G-protein coupled receptors. Selective activation of DREADDs via clozapine N-oxide (CNO) or compound-21 (C21) can produce excitatory effects through Gq (hM3Dq) and Gs (GsD) pathways which increase calcium and cyclic adenosine monophosphate (cAMP), respectively. Alternatively, inhibitory effects can be achieved using Gi (hM4Di) signaling, which reduces cAMP levels. Optogenetics (**B**) utilizes light-activated rhodopsin channels which can produce excitatory effects through sodium currents (Channelrhodopsin; ChR2) or inhibitory effects through chloride currents (Halorhodopsin; NpHR). Excitatory or inhibitory effects can also be achieved through light-activated Gq, Gs, or Gi signaling (OptoXR)

Chemogenetics involves using chemically engineered ligands and genetically-modified receptors [Designer Receptors Exclusively Activated by Designer Drugs (DREADDs) or ligand-gated ion channels (Pharmacologically Selective Actuator Modules, PSAMs)] to control cell signaling (Fig. 4a) [43]. Compounds such as clozapine-N-oxide (CNO), compound-21 (DREADD agonist 21), and perlapine activate hM3Dq (coupled with G_αq_ protein) and GsD (coupled with G_αs_ protein) so that they increase intracellular Ca^2+^ level [44] or activate adenylyl cyclase [45], respectively. However, the binding of DREADD agonist to hM4Di (coupled with G_αi_ protein) inhibits adenylyl cyclase [44]. Additionally, the activation of human κ-opioid receptor couples to G_αi_ (KORD) by the inactive drug-like metabolite salvinorin B (SALB) also leads to inhibit adenylyl cyclase [46]. As the non-canonical G protein signaling, CNO-driven activation of Rq(R165L) (alternative name: rM3Darr) facilitates intracellular arrestin-2/3 (β-arrestin) signaling [47]. When it comes to chemogenetics with chimeric ion channels, binding of pharmacologically selective effector molecules (PSEMs; ex, analogs of varenicline) to PSAMs directly increases Na^+^ (PSAM-serotonin receptor 5HT3 for activators) and Cl^−^ (PSAM-glycine receptor GlyR for silencers) influx [48].

Optogenetics is another powerful tool that is a light-related biological technique with invasive optical fibers implanted into the target brain region. At a specific time point, light stimulation dynamically changes the activities of cells which express light-sensitive receptors (opsin): channelrhodopsin (ChR), halorhodopsin (NpHR), archaerhodopsin (Arch), and OptoXR (Fig. 4b). ChR, a blue color-sensitive ion channel, has two subtypes depending on light-driven cellular influx of positive or negative charged ion. Light-driven opening of classical ChR (ex, ChR2) increases intracellular cations including Na^+^ ion and cellular excitability [49]. On the other hand, recently developed anion-conducting ChR (ACR) increases intracellular Cl^−^ ion for cellular inhibition [50]. Unlike blue light-sensitive inhibitory opsin ACR, NpHR is an orange light-sensitive inhibitory chloride pump [51]. Using different wavelengths of light, thus, ChR and NpHR together enable optical activation and silencing in the same cells [52, 53]. Arch is a blue-green light-gated proton pump so that its activation increases extracellular H^+^ ion levels and reduces cellular activities [54]. OptoXR is a green light-sensitive metabotropic receptor. According to the origins of chimeric receptors, OptoXR includes Opto A1 (from G_aq_-coupled α_1_ adrenergic receptor), B2 (from G_as_-coupled β_2_ adrenergic receptor), D1 (from G_as_-coupled D_1_ dopamine receptor), and A2AR (from G_as_-coupled adenosine A_2A_ receptor) [55–57].

Interestingly, neuromodulation techniques such as deep brain stimulation (DBS) are already being used in clinics to treat a variety of disorders including Parkinson’s disease and Tourette syndrome, as well as depressive disorder and obsessive-compulsive disorder [58]. Optogenetics and chemogenetics may have direct therapeutic uses in the future, allowing for more targeted neuromodulation in patients with psychiatric disorders. Although brain cell- and circuit-specific neuromodulation techniques have been revolutionary tools in the lab, it is necessary to minimize brain damage for potential clinical use. In particular, advances in optogenetics have utilized ultra-light-sensitive optogenetic modulator [59] and visible light-emitting nanoparticles with near-infrared light [60] to allow noninvasive penetration into deep brain tissue without optic fiber implantation. Therefore, continually developed future neuro-technologies will enable us to provide a key for treating psychiatric diseases.

## Conclusions

While these new technologies have allowed us to rapidly advance our understanding of psychiatric disorders in rodent models at the cellular, molecular, and brain circuit levels as summarized in Table 1, there remains a disconnect between our available knowledge, and effective treatments for patients. Given the vast complexity of cell types, gene expression profiles, and connections in the brain, it is highly unlikely that any single method will be a cure-all for psychiatric disorders. New treatments will be facilitated by a multitude of preclinical and clinical studies characterizing which risk gene variants have altered expression or function in which cell-types, how these specific cells are connected and influence neural activity within and between certain brain regions, and ultimately how this leads to pathological brain states and behavior.

**Table 1:** Overview of the advantages, disadvantages, and references to promising genetic, neuroimaging, and neuromodulation technologies

	Advantages	Disadvantages	References
**Genetics:**			
scRNA-seq	single cellular resolution	Expensive Large amount of data Improving bioinformatic pipelines	Kolodziejczyk et al. (2015) Ofengeim et al. (2017) Saliba et al. (2014)
CRISPR/Cas9	Cost and time efficient Precise editing Multiple genes simultaneously	Off-target effects Editing efficiency	Doudna & Charpentier (2014)
**Neuroimaging:**			
Fiber Photometry	Cell-type specific High temporal resolution	Lack of individual cellular information	Dana et al. (2015) Li et al. (2019)
1/2-Photon Imaging	Cell-type specific High temporal resolution High spatial resolution	Larger head mount Limited free movement Widely invasive surgery	Leinweber et al. (2014) Ozbay et al. (2018)
**Neuromodulation:**			
Optogenetics	Cell-type specific High temporal specificity Capability of fine temporal manipulation	Difficulty targeting deeper structures Implanted fiber cable	Boyden et al. (2005) Govorunova et al. (2015)
Chemogenetics	Cell-type specific Free moving behavior Higher penetrance Long duration effect	Potential off target effects of ligand Relative low temporal specificity	Armbruster et al. (2007) Magnus et al. (2019)

While this review primarily focused on recent molecular profiling, and neural imaging and modulation techniques, the importance of representative behavioral paradigms and animal models cannot be overemphasized for the validity of translational psychiatric research. Given the often comprehensive classifications of psychiatric disorders solely based on symptomology, rodent models have often been limited to representing certain aspects of mental disorders based on relatively subjective inferences about the emotional state of animals and their likeness to human conditions. Having said that, a new emphasis on advancing traditional behavioral models will be required to further gleam the emotional and cognitive state of animals. While rodents show common behavioral patterns with other mammals, including humans, it is also important to consider species-specific behaviors, as more natural selective pressures may be optimal for understanding unique behaviors for each species. This will be helpful for generalizing animal behavior and translating it to decipher the molecular basis of human psychiatric disorders. Further, improved nosology for clear behavior outcome measures and discovery of new biomarkers will also greatly advance our ability to create valid and representative rodent models, while simultaneously improving criteria for diagnosis and treatment evaluation.

Altogether, despite numerous examples of failed treatments that showed promise in preclinical studies, rodent models will be essential to advance both the understanding and treatment of psychiatric disorders. Used in conjunction with advancing technologies and improved clinical practice, these models will accelerate innovation in the field and have the potential to help improve the lives of the almost one billion individuals across the world, living with mental or substance use disorders.

## Data Availability

Not applicable.
